# The Changing Role of Health Care Professionals in Nursing Homes: A Systematic Literature Review of a Decade of Change

**DOI:** 10.3389/fpsyg.2017.02008

**Published:** 2017-11-14

**Authors:** Arend R. van Stenis, Jessica van Wingerden, Isolde Kolkhuis Tanke

**Affiliations:** ^1^Schouten Global, Centre of Research, Knowledge and Innovation, Zaltbommel, Netherlands; ^2^Faculty of Social Sciences, Institute for Psychology, Erasmus University Rotterdam, Rotterdam, Netherlands; ^3^Faculty of Social and Behavioural Sciences, Institute for Education & Pedagogy, Utrecht University, Utrecht, Netherlands

**Keywords:** changing role, skills, competences, health care professional, literature review, meaningful work, nursing homes

## Abstract

Although the role of health care professionals is known to have changed over the last years, few formal efforts have been made to examine this change through means of a scientific review. Therefore, the goal of this paper was to investigate the changing role of health care professionals in nursing homes, as well as the conditions that make this change possible. A systematic review of health care literature published in the last decade (2007–2017) was utilized to address these goals. Our findings suggest that although health care in nursing homes is shifting from task-oriented care to relation-oriented care (e.g., through an increased focus on patient dignity), various obstacles (e.g., negative self-image, work pressure, and a lack of developmental opportunities), needs (e.g., shared values, personal development, personal empowerment, team development, and demonstrating expertise), and competences (e.g., communication skills, attentiveness, negotiation skills, flexibility, teamwork, expertise, and coaching and leadership skills) still need to be addressed in order to successfully facilitate this change. As such, this paper provides various implications for health care research, health care institutions, practitioners, HR professionals and managers, and occupational health research.

In recent years, there's been a continuing interest in the role of health care professionals in nursing homes (e.g., Hasson et al., 2008; Huizenga et al., 2016; Thompson et al., 2016). Although the profession in itself is by no means entirely novel, some research suggests that the role of the health care professional in nursing homes may have been changing over the last few years (e.g., Huizenga et al., 2016). If changes such as these were to be applied on a national scale, this could have major implications for not only the patients receiving care, but also for the health care professionals themselves. For instance, the adaptation of such new roles could require new sets of skills, or could bring about its own obstacles during implementation. Indeed, recent research suggests that current nurse competences in nursing homes may be insufficient to deal with the expected role transitions (Bing-Jonsson et al., 2016). In addition, there may be other factors that prevent these changes from becoming apparent in practice.

An overview of the changes currently occurring within nursing homes would allow the health care sector to alter its strategies accordingly and deal with the lack of competence. However, so far, no formal attempts have been made to fully explore the changing role of the health care professional in nursing homes, nor the circumstances this change would depend on. Therefore, the aim of the current study was two-fold: (i) To investigate the changing role of health care professionals in nursing homes, and (ii) to investigate the conditions that make this change possible. Through means of a systematic review of the health care literature, we set out to address these goals by discussing the current changes in nursing home roles, the caregiver needs related to these changes, and the caregiver skills and competences required for these changes. Based on our research, we propose various implications for both health care professionals and health care institutions, as well as suggestions for future research.

## Method

### Search strategy

**Figure 1:**
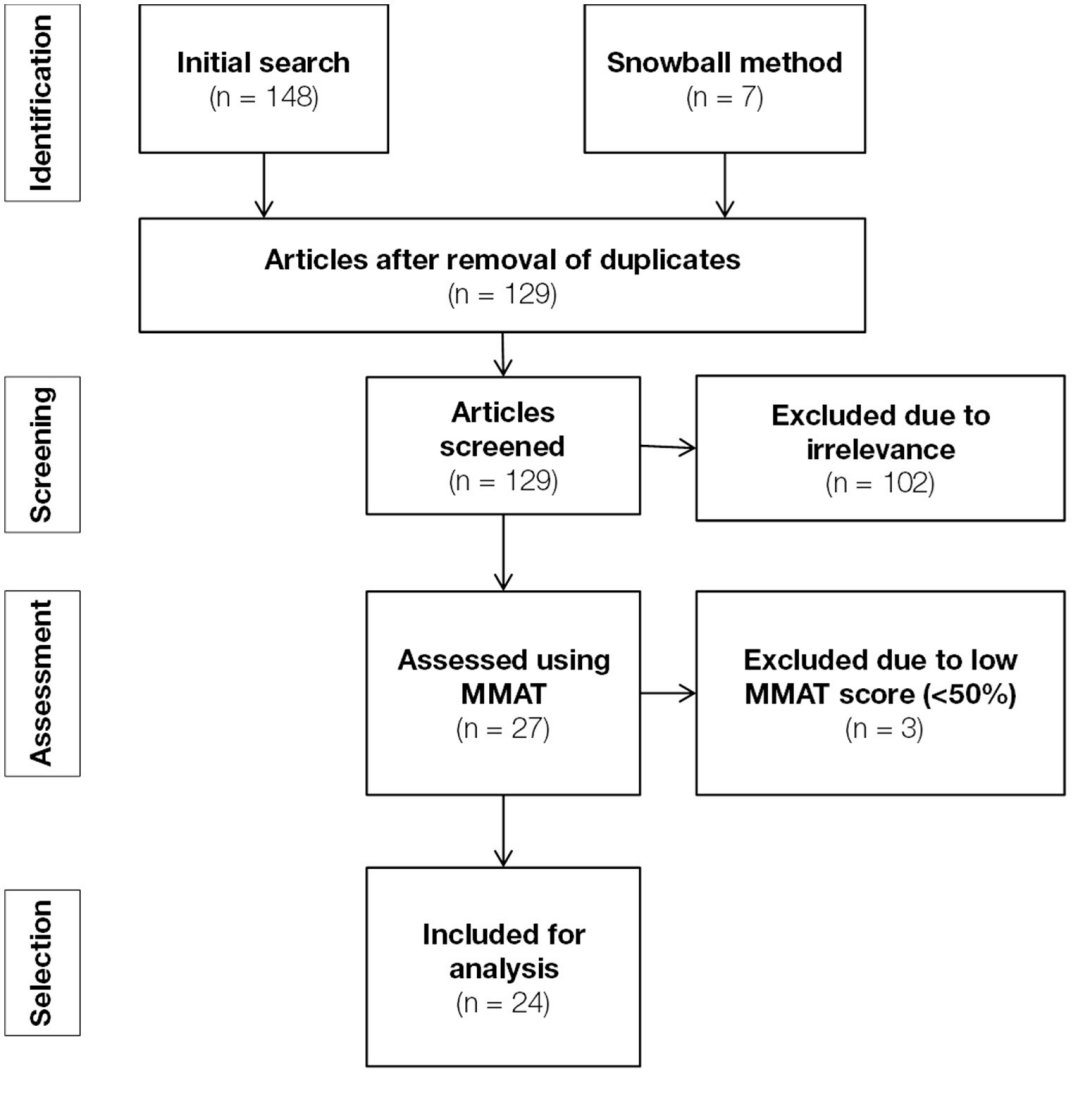
Flowchart summarizing the various stages of literature search and selection.

A systematic literature search was conducted using three databases: Scopus, PubMed, and Web of Science. The health care literature was searched using the search terms determined by the authors, which are summarized in Table 1. We limited our search so as to only include findings that focus on the circumstances (row 4) that are related to the changing roles (row 5) of caregivers (row 3) working in diverse forms of care (rows 1 and 2). In order to limit the amount of results and increase the relative amount of relevant results, rows 3 and 5 were limited to searching by title only. In addition, in order to maintain the actuality of this review, we only included articles that were published between January 2007 en March 2017. The initial search yielded 148 findings (see Table 2; Figure 1). After removal of duplicates, 122 findings remained for further quality assessment.

**Table 1:** Summary of the search terms used in scopus, pubmed, and web of science to provide the initial literature search.

**Row**		**Search terms**	**Search restrictions^a^**
1		((nursing OR care OR retirement OR residential) AND home) OR “aged care facilit^*^” OR “for the aged” OR “assisted living” OR “care institution” OR institutional	Article title, Abstract, Keywords (Scopus)/All Fields (PubMed)/Topic (WoS^b^)
2	And	((elderly OR “long^*^term” OR senior OR “older people”) AND care)	Article title, Abstract, Keywords (Scopus)/All Fields (PubMed)/Topic (WoS)
3	And	Caretaker OR caregiver OR nurse OR “health^*^ professional” OR “^*^care professional” OR staff	Title only (all search engines)
4	And	Factor OR determinant OR antecedent OR development^*^ OR requirements OR necessities OR correlate OR change OR changing OR culture	Article title, Abstract, Keywords (Scopus)/All Fields (PubMed)/Topic (WoS)
5	And	Role OR portrayal OR competenc^*^ OR skill OR abilit^*^ OR characteristics OR expertise OR proficienc^*^ OR capabilit^*^ OR capacit^*^ OR “meaning” OR “meaningful” OR “meaningfulness”	Title only (all search engines)

a *All searches were limited to findings published between 2007 and 2017*.

b *Web of Science*.

**Table 2:** The number of studies found per source across the various stages of the literature selection process.

**Stage**	**Source**	**Remaining number of findings**
Initial findings		
	Scopus	55
	PubMed	36
	Web of Science	57
	Total	148
Removal of duplicates		
	Scopus	54
	PubMed	29
	Web of Science	39
	Total	122 (26 duplicates removed)
Initial screening		
	Total	20 (102 excluded)
Snowball method		
	Total	27 (7 included)
In-depth quality assessment		
	Total	24 (3 excluded)

### Quality assessment

Methodological quality was examined using the Mixed Methods Appraisal Tool (MATT; Pluye et al., 2011). The tool provides a checklist that can be used to assess the quality of quantitative, qualitative and mixed methods studies (Pluye and Hong, 2014). Compared to other tools, the MMAT specifically includes criteria for appraising mixed methods studies. The MMAT is known to be a reliable and efficient tool to assess whether a study is suitable for systematic literature search (Pace et al., 2012). As such, the quality of the initial findings was assessed according to the criteria defined by the MMAT (see Appendix).

The remaining 122 studies were screened based on the relevance of their research questions and the degree to which these studies' answered their research questions. This was done by screening the titles and abstracts of these studies. Studies that did not address our research questions (i.e., studies focusing on topics other than the role of health care professionals in nursing homes) or were unfindable, were excluded from further analysis. Based on these criteria, 102 studies were excluded after the first screening, leaving 20 studies for the next phase of the procedure (see Table 2). In order to extend the literature search, the snowball method was subsequently applied to the results of the initial screening. This means we searched for articles that were referenced by the findings of the initial screening. Only studies that were deemed relevant to our research question were maintained for further analysis. This was again assessed by screening the titles and abstracts of said studies. This additional search action yielded 7 additional results, which means 27 studies remained for in-depth quality assessment. The quality of the 27 remaining studies was further assessed by answering design-specific questions about the methodological quality of these studies, as defined by the MMAT procedure (Pluye et al., 2011). Studies with a low MMAT score (< 50%) were subsequently removed. This caused another 3 studies to be excluded, leaving 24 articles for the final analysis (see Tables 3, 4).

**Table 3:** General description of sources included in this review, including author names, year, study design, sample characteristics, measures, and the main findings.

**#**	**References**	**Study design**	**Sample characteristics**	**Measure(s)**	**Main finding(s)**
1	Adra et al., 2015	Qualitative: Qualitative description	Residents of nursing homes (*n* = 20), nurses (*n* = 11), and family caregivers (*n* = 8) in Lebanon.	Interviews.	Four themes were identified: 1) Maintaining family connectedness, 2) engaging in worthwhile activities, 3) maintaining and developing significant relationships, and 4) holding and practicing spiritual beliefs.
2	Arnetz and Hasson, 2007	Quantitative: Non-randomized controlled Intervention: Educational toolbox intervention	Nursing staff from two municipalities in western Sweden. Intervention group: *n* = 213–270. Reference group: *n* = 606–647.	Quality-work-competence (QWC). Pyramid quality of care questionnaire. Relative questionnaire.	The intervention group showed a larger significant improvement for some aspects of self-rated knowledge and psychosocial work environment than the reference group.
3	Bing-Jonsson et al., 2014	Mixed method: Sequential exploratory Quantitative: Descriptive study Qualitative: Qualitative description	Experts (about older people care) from Norway. *N* = 42.	Interviews. Nursing Older People—Competence Evaluation Tool (NOP-CET).	Various important aspects of competences were identified, such as health promotion, disease prevention, treatment, palliative care, ethics and regulation, assessment and taking action, covering basic needs, communication and documentation, responsibility and activeness, cooperation, and attitudes toward older people.
4	Bing-Jonsson et al., 2016	Quantitative: Descriptive study	Nursing staff in Norway. *N* = 1016.	Nursing Older People—Competence Evaluation Tool (NOP-CET).	The participants expressed competence in all variables measured. However, the degree to which this was the case varied.
5	Blomberg et al., 2013	Qualitative: Qualitative description	Healthcare professionals in Sweden. *N* = 13.	Interviews.	The meaning of work has changed over time from a focus on obstacles to one of opportunities.
6	Burger et al., 2009	Qualitative: Descriptive study	Expert panel in the USA. *N* = 31.	Focus group interview.	There's a shift to resident-focused care. Nurses should be involved in decision making and be empowered. Various themes for the new roles of registered nurses were identified, including: Autonomy, dignity, respect, flexibility, leadership, professional development, and considerate behavior. Nurses currently experience high work pressure and are not adequately prepared for this new work role.
7	Cairns et al., 2013	Quantitative: Descriptive study	Health and social care professionals across four NHS Trusts in England. *N* = 192.	Dignity questionnaire.	Dignified care was described with terms such as: “respect,” “being treated as an individual,” “being involved in decision making,” and “privacy.” The most important aspects were “being treated as an individual” and “maintaining privacy.” Relational components were more important for dignified care than physical caring tasks.
8	DeHart et al., 2009	Qualitative: Descriptive study	Nursing home staff, policy makers, and related professionals in the USA. *N* > 20.	Interviews.	Assistants often lack the ability to deal with conflicts. Suggested competences include: Communication with a focus on improving relations, self-reflection, realizing the dependence of residents, client-oriented care, individualized care, sharing of values, teamwork.
9	Duffy et al., 2009	Quantitative: Descriptive study	Staff working in Continuing Care homes for older people with dementia in the UK. *N* = 61.	Maslach Burnout Inventory (MBI). Jeffcott Reciprocity Questionnaire. Inventory of Geriatric Nursing Self-Efficacy. Occupational Commitment Questionnaire (OCQ).	Burnout was most strongly predicted by (a lack of) self-efficacy. Other factors include reciprocity, occupational commitment, and demographic factors.
10	Ellis and Rawson, 2015	Qualitative: Qualitative description	Registered nurses, nurses in training, and assistants in Australia. *N* = 20.	Interviews.	Two themes were identified: “What it's like for them”: awareness of the consequences of relocation, staffing and nursing care; additional services and the environment, “We can make it better”: suggestions for improving relocation, e.g., socializing with new residents, and the importance of person-centered care.
11	Engström et al., 2011	Quantitative: Cross-sectional analytic	Caregivers in elderly care in Sweden. Formal competence: *n* = 447. No formal competence: *n* = 125.	Satisfaction with Work Questionnaire (SWQ). Conditions of Work Effectiveness Questionnaire II (CWEQ II).	Those without formal competence experienced a relatively higher workload, more communication obstacles, less competence, poorer sleep and more stress than those with formal competence.
12	Engström et al., 2010	Quantitative: Non-randomized controlled Intervention: Training program consisting of 8 group sessions during 9 months.	Female caregivers in Sweden. Intervention: *n* = 14. Control: *n* = 32.	Spreitzer's Empowerment scale. Psychosocial aspects of job satisfaction.	Over time, the amount of “criticism” significantly increased for the intervention group, whereas the control group remained the same. Empowerment showed a positive correlation with most aspects job satisfaction.
13	From et al., 2013	Quantitative: Cross-sectional analytic	Staff from 14 communities in Sweden. Nursing assistants: *n* = 70. Enrolled nurses: *n* = 163. Registered nurses: *n* = 198.	Quality of care from the Patients' Perspective (QPP). The Creative Climate Questionnaire (CCQ). Stress of conscience questionnaire (SCQ). Questionnaire on education and competence development. Health Index (HI).	Work-related competences were better developed than social competences. The most important competence was deemed to be work-related in nature. The culture was creative in some regards, but stagnant in others. Well-being of nurses was generally good, but registered nurses scored worse compared to the other types of nurses. Education is often utilized to make one feel “safe” at work, and is more often (voluntarily) applied in practice by registered nurses than the other nurses.
14	Ha et al., 2014	Quantitative: Descriptive study	Care workers in 14 nursing homes in Korea. *N* = 504.	5-point Likert scales measuring various variables defined by other studies.	High-performance partially mediated turnover intention through organizational support and commitment. Turnover intention was influenced the most by organizational commitment.
15	Hasson et al., 2008	Qualtitative: Descriptive study	Link nurses from 10 nursing homes in Northern Ireland. *N* = 14.	3 focus group interviews.	Although link nurses have the potential to improve palliative care they experienced a number of difficulties, such as lack of managerial support, a transient workforce and a lack of adequate preparation. Favorable conditions included external support, monthly meetings, access to resource files and peer support.
16	Hasson and Arnetz, 2008	Quantitative: Cross-sectional analytic	Nursing staff in two older people care organizations in Sweden. Home care: *n* = 298. Nursing homes: *n* = 565.	Quality-work-competence (QWC). Single-item work satisfaction rating.	Home care staff had insufficient knowledge, but experienced less strain compared to nursing homes' staff. Both care settings were equal in terms of exhaustion, mental energy and work satisfaction. Exhaustion was the strongest negative predictor of work satisfaction.
17	Huizenga et al., 2016	Qualitative: Qualitative description	Registered nurses in geriatrics and gerontology in The Netherlands, both working in nursing homes as well as in home care, general healthcare, hospitals, etc. *N* = 67.	7 focus group interviews.	Although nurses often fulfill all of the “CanMEDS” roles, they rarely possess all the required competences. Having fewer patient activities correlates with a lower expression of competences such as social networks; design; research; innovation of care; legal, financial and organizational frameworks; professional ethics and professional innovation.
18	Kinnear et al., 2014	Qualitative: Qualitative description	Healthcare professionals (*n* = 48) and social care professionals (*n* = 33) in England.	8 focus group interviews.	Dignity is considered to be a central aspect of care, and it encompasses a focus on “the little things,” as well as creating a safe atmosphere and treating one another as equals and individuals.
19	Rehnsfeldt et al., 2014	Qualitative: Qualitative description	Relatives of elders receiving care in Norway, Denmark, and Sweden. *N* = 28.	Interviews.	Dignity encompasses “feeling at home” and “the little extras.” However, non-caring cultures focus on routine, efficiency, and instrumentalism.
20	Rodríguez-Martín et al., 2016	Quantitative: Descriptive study	Nursing staff working in 62 units for older people in Southwest Finland. *N* = 874.	Individualized Care Scale-Nurse-B (ICS-Nurse-B). Questionnaire for nurses' socio-demographic and organizational data.	Participants generally had positive perceptions about the amount of individualized care for older people, which included taking into account patients' clinical situations and patients' decisional control. Individualized care provision correlated positively with age and type of organization.
21	Thompson et al., 2016	Qualitative: Qualitative description	Nursing staff in Great Britain. *N* = 13.	5 interviews.	Economic policies and the nature of nursing work were though to negatively impact the occupational status of nurses. This in turn influenced nurses' perception of their roles and their ability to enact their roles.
22	Van der Kooij et al., 2013	Quantitative: Randomized controlled trial Intervention: Integrated emotion-oriented care	Professional caregivers in 16 psychogeriatric nursing home wards in 14 nursing homes in The Netherlands. Experimental group: *n* = 46. Control group: *n* = 53.	Emotion-oriented Skills in the Interaction with Elderly People with Dementia (ESID). 39 criteria for quality standards for usual nursing home care.	Integrated emotion-oriented care increased caregivers' emotion-oriented skills and knowledge of residents, and did not consume more time than traditional care.
23	Wilson and Davies, 2009	Qualtitative: Descriptive study	Residents (*n* = 16), staff (*n* = 25), and families of residents (*n* = 18) from three care homes in England.	8 focus group interviews.	Staff adopted individualized task-centered, resident-centered and relationship-centered approaches to care delivery, which in turn influenced what relations where developed between residents, families, and staff.
24	Yeatts and Cready, 2007	Mixed method: Sequential exploratory Quantitative: Cohort study Qualitative: Qualitative description	Certified nurse aides (CNA, *n* = 314–354), nurses (*n* = 149–164), and residents of family members (*n* = 530–578) from 10 nursing homes in the USA.	Custom survey to measure global empowerment and its dimensions of autonomy, impact or meaningfulness, and competence, rating of CNA performance, self-esteem, burnout, job satisfaction, satisfaction with scheduling, commitment, intent to quit, and absenteeism. Observations of over 270 certified nurse aides meetings. Examination of weekly team-meeting summaries. Examination of written weekly responses and requests from nurse management.	Having work teams improved CNA empowerment; CAN performance; resident care and choices; procedures, coordination, and cooperation between CNAs and nurses; and tentatively decreased turnover. Work attitudes showed mixed results.

**Table 4:** Summary of confirmed MMAT-criteria and final MMAT-scores per source.

**#**	**References**	**Confirmed MMAT-Criteria**	**Final MMAT-Score**
1	Adra et al., 2015	1.1 + 1.2 + 1.3	75% (ql)
2	Arnetz and Hasson, 2007	3.3 + 3.4	50% (qn)
3	Bing-Jonsson et al., 2014	1.1 + 1.2 + 4.1 + 4.3 + 4.4 + 5.1 + 5.2	50% (ql) 75% (qn) 66.6% (mm)
4	Bing-Jonsson et al., 2016	4.1 + 4.2 + 4.3	75% (qn)
5	Blomberg et al., 2013	1.2 + 1.3	50% (ql)
6	Burger et al., 2009	1.2 + 1.3	50% (ql)
7	Cairns et al., 2013	4.1 + 4.2	50% (qn)
8	DeHart et al., 2009	1.2 + 1.3	50% (ql)
9	Duffy et al., 2009	4.1 + 4.3	50% (qn)
10	Ellis and Rawson, 2015	1.1 + 1.2 + 1.3	75% (ql)
11	Engström et al., 2011	3.2 + 3.3 + 3.4	75% (qn)
12	Engström et al., 2010	3.2 + 3.4	50% (ql)
13	From et al., 2013	3.1 + 3.2 + 3.3	75% (qn)
14	Ha et al., 2014	4.3 + 4.4	50% (qn)
15	Hasson et al., 2008	1.1 + 1.2 + 1.3	75% (ql)
16	Hasson and Arnetz, 2008	4.2 + 4.4	50% (qn)
17	Huizenga et al., 2016	1.2 + 1.4	50% (ql)
18	Kinnear et al., 2014	1.1 + 1.2 + 1.3	75% (ql)
19	Rehnsfeldt et al., 2014	1.2 + 1.4	50% (ql)
20	Rodríguez-Martín et al., 2016	4.1 + 4.2 + 4.3	75% (qn)
21	Thompson et al., 2016	1.2 + 1.3 + 1.4	75% (ql)
22	Van der Kooij et al., 2013	2.1 + 2.3	50% (qn)
23	Wilson and Davies, 2009	1.1 + 1.2 + 1.3 + 1.4	100% (ql)
24	Yeatts and Cready, 2007	1.1 + 1.2 + 3.2 + 3.3 + 3.4 + 5.1 + 5.2	50% (ql) 75% (qn) 66.6% (mm)

*ql, qualitative; qn, quantitative; mm, mixed method*.

### Analysis strategy

In order to answer our research questions and make findings comparable, we generated an overview including research designs, sample characteristics, measurements, and main findings for the 24 studies that remained after quality assessment (see Table 3). Based on this overview, we set out to identify the main themes within the assessed literature by scanning these findings for similarities.

## Results

The search resulted in 24 articles. An overview of these sources can be found in Table 3. All studies were clustered based on the following themes: Changes in nursing home roles, caregiver needs relating to role transitions, caregiver skills and competences.

### Changes in nursing home roles

#### Relation-oriented care

A common theme in the observed literature is the shift from task-oriented care to relation-oriented care. Whereas health care institutions would previously focus primarily on performance-driven standardization of procedures, they are now also concerned about the quality of individualized care for patients (Burger et al., 2009; Cairns et al., 2013; Kinnear et al., 2014; Adra et al., 2015; Rodríguez-Martín et al., 2016). This shift in focus may well be due to the negative press reports that have criticized healthcare for being too impersonal over the last few years (e.g., Cairns et al., 2013; Kinnear et al., 2014).

Various sources suggest that maintaining personal relationships with patients has become an important part of caregiving (e.g., Kinnear et al., 2014; Adra et al., 2015). Although it is sometimes thought that these type of relationships develop naturally through daily care, Wilson and Davies (2009) suggest that the development of the type of relationship is actually controlled by the caregivers themselves. Specifically, they state that three types of relationships can be developed: (1) Individualized and task-centered, (2) patient-centered, and (3) relationship-centered, with the last two yielding the most positive experiences for care receivers and their families. The first type focuses only on completing tasks pragmatically, the second one takes the needs of the care receiver into account, and the third type takes into account the needs of both the care receiver and other involved parties, such as family and the rest of the community. It is said that the relationship-centered approach requires caregivers to actively connect the various parties involved in the caregiving community (Burger et al., 2009; Wilson and Davies, 2009; Adra et al., 2015). However, according to Adra et al. (2015), this is often still not the case in practice. Thus, part of nurses' new relation-oriented role is to actively pursue a relationship-centered approach of healthcare by bringing the various parties involved together.

Another important aspect of relation-oriented care is *dignity*, which involves treating care receivers with respect, focusing on “the little things” in life, and making patients feel “at home” (Cairns et al., 2013; Kinnear et al., 2014; Rehnsfeldt et al., 2014). An example of this can be found in the research of Ellis and Rawson (2015), who examined the importance of personalized care for elderly people moving into nursing homes. Their results demonstrated that caregivers could increase the perceived feelings of dignity by getting to know the patients on a personal level at the start of their residency. In addition, Cairns et al. (2013) concluded that health care professionals considered relational components of care to be more important for dignified care than physical caring tasks.

Relationship-centered care and dignity aren't entirely new concepts, though, and can already be seen in various existing roles in healthcare, such as the roles described by Huizenga et al. (2016): The “communicator,” “collaborator,” and “manager.” The communicator is defined as being responsible for maintaining personal relations with the involved parties, with an emphasis on openness and empathy. The collaborator focuses on facilitating cooperation between various disciplines within healthcare to achieve optimal patient care. The manager lastly coordinates the developments affecting patients, and prevents fragmentation of care (Huizenga et al., 2016). This demonstrates that at least some of the roles health care professionals fulfill already contain elements of relation-oriented care, but not all of them.

All in all, the new role of health care professionals can best be summarized as one consisting of a relation-oriented approach to care, which involves connecting parties with one another and giving care receivers a sense of “dignity.”

#### Benefits of relation-oriented care

Few researchers have explicitly examined the benefits of the shift in focus toward relation-oriented care. Only one study by Wilson and Davies (2009) suggests that residents of nursing homes express more positive feelings with relation-oriented care compared with task-oriented care. However, there are other sources that suggest that specific aspects of relation-oriented care, such as dignity, may also be worthwhile to develop. For example, Adra et al. (2015) suggest that if caregivers understand their patients' needs emotions, they will also be able to better match the care they provide with their patients. This means that there is at least some evidence that suggests relation-oriented care is actually beneficial, which may provide further incentive to increase the amount of relation-oriented care in practice.

#### Obstacles in relation-oriented care

Although previous research findings suggest the shift to relation-oriented care can be considered to be a positive one, there appear to be multiple obstacles that prevent this shift from fully being implemented in all parts of healthcare. For instance, Thompson et al. (2016) suggest that a lack of economical investments, combined with negative public perceptions of health care professions (e.g., “low-skilled work”), negatively influence caregivers' own confidence in their ability to fulfill their roles effectively. In addition, since patients often have to pay for their own health care, this causes them to view their caregivers as service providers, rather than medical experts. This further hinders caregivers' ability to form a personal relationship, as it is now seen as a commercial transaction rather than an interpersonal relation. The authors also mention that routine work may prevent health care professionals from showing their true expertise. This in turn may again negatively influence their own self-image, which prevents them from forming optimal relationships with patients (Thompson et al., 2016). Thus, it seems that the negative image surrounding health care professionals may inhibit them from developing meaningful relations with patients.

In addition to image-related obstacles, many authors also highlight high work pressure as a potential obstacle for relation-oriented care (e.g., Hasson et al., 2008; Wilson and Davies, 2009; Cairns et al., 2013; Ha et al., 2014; Kinnear et al., 2014; Adra et al., 2015; Huizenga et al., 2016). A high workload means that caregivers have less time to invest into a meaningful relationship with their patients (Kinnear et al., 2014; Adra et al., 2015). It should be noted that caregivers often see relationship building as an additional burden, even though this is actually inherent to the work itself (Wilson and Davies, 2009; Cairns et al., 2013; Kinnear et al., 2014). This suggests that the work itself may not need to be changed to allow for relation-oriented care, but instead, caregivers' perception about the meaning of their work should be changed, which could be addressed by relationship-centered dialogue (e.g., Burger et al., 2009; Wilson and Davies, 2009; Adra et al., 2015).

One final obstacle has to do with a lack of education and developmental opportunities in health care. For example, research by Engström et al. (2011) demonstrated that caregivers without formal education showed higher levels of perceived workload, communication issues, sleep disturbance, and stress-related issues, as well as lower feelings of competence compared to colleagues with formal education. Similarly, research by Duffy et al. (2009) has shown that lower levels of self-efficacy predicts higher levels of burn-out for health care professionals. Lastly, Burger et al. (2009) suggest caregivers may feel incompetent in the light of organizational culture shifts, as this may require new skills that caregivers have not yet acquired. As such, a lack of education implies caregivers won't have the required skills or confidence to successfully build up a relation-oriented relationship with patients.

In sum, research points out three major obstacles that can prevent the development of relation-oriented relationships in health care: Bad image among caregivers, high workload, and a lack of developmental opportunities.

### Caregiver needs relating to role transitions

During our search, various themes arose regarding the needs of caregivers relating to their role transitions. These are the needs for shared values, personal development, personal empowerment, team development, and demonstrating expertise.

#### Need for shared values

In their article about meaningful work in elder care, Blomberg et al. (2013) stress the importance of sharing and reflecting upon caregivers' personal values and convictions in order to improve their relations with clients and family, an idea which is echoed by various other researchers (e.g., Wilson and Davies, 2009). Rather than being an individual matter, the need for shared values is actually considered to be a culture-related issue, which implies that the health care organization as a whole should promote a culture of shared values and meaningful work in order to improve caregivers' relationships with clients and other third parties (DeHart et al., 2009; Rehnsfeldt et al., 2014). Thus, in order to make role transitions possible, it appears the need for shared values should be addressed first.

#### Need for personal development

Although caregivers often want to feel competent at work in order to fulfill their roles effectively (Burger et al., 2009; Duffy et al., 2009; Engström et al., 2011), oftentimes the need for development is not met by health care institutions (Hasson and Arnetz, 2008; Engström et al., 2011; From et al., 2013). Developing caregivers' competences is thought to be an important part of role transitions, as it will add to their feelings of self-efficacy (From et al., 2013). In addition, rather than offering single training efforts, it seems best for health care institutions to offer continuous developmental opportunities to actually improve health care (From et al., 2013; Thompson et al., 2016). Thus, caregivers' need for development should be addressed in order to allow for better role transitions.

#### Need for personal empowerment

Oftentimes, caregivers aren't given the opportunity to be involved in decision-making processes related to personal and organizational change. This lack of empowerment causes them to lose sight of their ability to control and change their own role within health care, leading to a lack of own initiatives for change (Burger et al., 2009). Increasing empowerment is known to improve caregivers' communication skills (Engström et al., 2010), which may help in creating new opportunities to take initiative. Focusing on opportunities, rather than obstacles, may aid in achieving a sense of empowerment among caregivers (Blomberg et al., 2013). As such, these results suggest providing a sense of empowerment is not only necessary, but also achievable.

#### Need for team development

Not only should individual caregivers be offered sufficient developmental opportunities, but according to various authors, teams in their entirety should be developed as well (Yeatts and Cready, 2007; Blomberg et al., 2013). One way of achieving this, is through the introduction of self-managing teams, which in addition to developing the team itself, also leads to increased feelings of autonomy, competence, and empowerment (Yeatts and Cready, 2007). Yeats and Cready also note that sufficient time should be made for self-managing teams to operate without sacrificing time spent on core caregiving tasks, and that managerial support is essential for team success. In that case, having self-managing teams could be a good way of creating more developmental opportunities for caregivers.

#### Need for demonstrating expertise

Both caregivers and their employers report that the roles of caregivers are often interpreted as encompassing fewer tasks than they actually entail (Huizenga et al., 2016; Thompson et al., 2016). Health care organizations often do not recognize the potential of caregivers, which leads caregivers to not recognize and act upon their own expertise (Huizenga et al., 2016). Work itself then becomes more routine-based, creating a knowledge gap when complex questions arise from clients (Thompson et al., 2016). Thus, caregivers' expertise should be acknowledged by organizations in order to further express said expertise.

### Caregiver skills and competences

Based on various sources, we were also able to compile a list of competences that are deemed necessary for caregivers in order to fulfill their (changing) roles effectively (Hasson and Arnetz, 2008; Burger et al., 2009; DeHart et al., 2009; Bing-Jonsson et al., 2014, 2016; Adra et al., 2015; Thompson et al., 2016). These include:

- Communication: Listening skills, providing feedback, discussing choices, discussing complaints, and building relationships in daily care.
- Attentiveness: Being open to clients' worries, picking up signals, and addressing emotions.
- Negotiation: Taking into account interests of those involved, facilitating conversations between those involved, making decisions after consideration, and solving conflicts.
- Flexibility: Observe and adjust to the needs of clients, and creative problem solving.
- Teamwork: Working with colleagues from diverse backgrounds, taking responsibility as a team member, daring to ask questions, supporting colleagues, complementing other colleagues' qualities.
- Expertise: Specific knowledge regarding health, palliative care, dementia, ethics, IT, use of medicine, etc.
- Coaching and leadership: Coaching colleagues, taking the initiative to exercise influence.

Although this list may not be exhaustive, it provides an extensive overview of various skills that may be useful in developing caregivers' roles.

#### Developing competences

Although many authors stress the importance of developing caregivers' competences, few authors have actually researched how this should be achieved specifically. However, some suggestions from intervention studies include having caregivers design their own learning trajectory, making sure learnt theory is immediately and effectively applicable to practice, and having co-workers (i.e., “internal experts”) train their fellow colleagues (Arnetz and Hasson, 2007; Hasson and Arnetz, 2008; Van der Kooij et al., 2013). It should be noted that internal experts should always be supported by other specialists and have sufficient knowledge about the subject, in addition to having didactic skills and having managerial support as well.

## Discussion

To our knowledge, this study presents one of the first systematic reviews of the changing role of health professionals in nursing homes. This study aimed to investigate the changing role of health care professionals in nursing homes, as well as the conditions that make this change possible. This was achieved by means of a systematic review of the health care literature, whereby the literature was sub-divided over three main themes: Changes in nursing home roles, caregiver needs relating to role transitions, caregiver skills and competences.

In terms of the first goal, we can conclude that the role health care professionals fulfill in nursing homes has shifted from standardized, task-oriented care to more individualized, relation-oriented care. For the health care professional, this means building up a mutual relationship with the care receiver and other third parties, for example through an increased emphasis on client dignity. Although various existing roles already take into account various aspects of client-oriented care, such as the roles of communicator, collaborator, and manager as defined by Huizenga et al. (2016), these and other roles will have to be further developed to allow for a complete shift toward relation-oriented care at some point in time.

However, despite the admitted importance of this change, and as an answer to our second research question, many caregivers still view the shift to relation-oriented care as a burden, as there are still various obstacles that prevent them from implementing this change (e.g., negative self-image, work pressure, and a lack of developmental opportunities). In addition, the literature specifies a variety of needs that need to be fulfilled in order to implement this change effectively, such as the needs for shared values, personal development, personal empowerment, team development, and demonstrating expertise. Lastly, the shift to relation-oriented care will also require various new competences, such as communication skills, attentiveness, negotiation skills, flexibility, teamwork, expertise, and coaching and leadership skills.

In short, although there is a general consensus that the role of the health care is shifting to be more relation-oriented, there are still various obstacles that need to be overcome, various needs that need to be fulfilled, and multiple competences that need to be developed in order to make this possible on a larger scale.

### Limitations

Although we tried to limit the amount of limitations as much as possible, there are still some factors that should still be kept in mind when interpreting the conclusions presented in this article. Firstly, we limited our scope to only include those articles that referred to a specific form of health care (i.e., nursing homes), rather than having a broader search strategy. It should be noted that despite this limitation, various aspects of nursing homes are indeed representative of other forms of care as well. Nonetheless, we urge readers to be cautious when generalizing these results to other parts of health care.

Secondly, in most of the studies examined in this review, the results were based mostly on self-reports of participants, rather than more “objective” measures. Although this is not something that was caused by our methodology specifically, it does mean that the results presented in this article should be interpreted with potential self-report bias in mind. However, since caregivers' own perceptions will most likely determine the course of health care development in the first place, these results remain valid nonetheless.

Thirdly, because of the rigor of our methodology, only a relatively small number of articles ended up being discussed in this study. However, since the final selection has been extensively screened beforehand in terms of methodological quality and relevance, we feel that the lack of quantity is mitigated by having a high-quality sample instead. Lastly, we included a relatively large amount of qualitative studies compared to quantitative studies, which may indicate a lack of quantitative support at first sight. However, since we did not filter based on study type (i.e., qualitative vs. quantitative), we feel that this research sample accurately reflects the current state of research.

Lastly, we did not make a distinction between various types of nursing homes that exist, such as government vs. private nursing homes. Although some authors did specify the type of nursing home investigated, many did not. However, we think this omission does not significantly impact the overall conclusions of our review.

### Implications

Our review has various implications for both theory and practice. In terms of theory, this study adds to the existing health care literature by being among the first to systematically examine the changing role of health professionals in nursing homes. By limiting the scope to the years 2007 to 2017, it provides a state-of-the-art overview that can be used as a base for future health care research. Furthermore, this study also adds to the literature on meaningful work by linking the concept of meaningful work to health care, which is directly relevant to the field of occupational (health) research. Lastly, our review provides a methodological framework that could be used in future research as a standardized method for investigating role changes in not just the health care sector, but other sectors as well.

In terms of practice, our review offers a concise overview of guidelines that can be used by health care institutions and individual caregivers to adequately prepare for the expected change in nursing home roles. Specifically, our review highlights the nature of the changing role of the health care professional in nursing homes, as well as the potential obstacles, needs, and required competences that need to be addressed in order to make the role transition as smooth as possible. HR professionals and managers working in health care could use this information to further improve their business and development strategies. For instance, the competences provided in our review could be used by HR professionals for the training and development of existing health care personnel, or even for the selection and assessment of new personnel. The obstacles and needs we highlight can be used by managers to generate a better understanding of the potential issues that individual caregivers might run into during their careers, as well as simultaneously providing general solutions to these issues. In addition, individual caregivers may use the information presented in this paper as a means of facilitating bottom-up feedback in health care institutions. More specifically, this may help caregivers give insight into their own concerns regarding their changing roles, which they can subsequently use to communicate these concerns to their superiors. In short, based on our results, we would recommend health care professionals and their managers to enter into dialogue with one other to discuss the needs and obstacles of caregivers. Having a mutual conversation will likely generate the best possible circumstances for creating smooth role transitions.

### Suggestions for future research

Considering our limitations and implications, various suggestions for future research can be made. Because our search was limited to only including health care research related to nursing homes, it makes sense to broaden this scope in future research by including other forms of health care as well. In addition, (quantitative) empirical research could focus on filling the gaps that were highlighted in the current study, such as the lack of objective measures and the lack of longitudinal research. Furthermore, it would be interesting to not only rely on self-reports of caregivers, but also reports from other parties, such as care receivers and their families. Lastly, it would be interesting to see whether or not our findings can be generalized to fields other than health care as well. Thus, future research could focus on using the methodological framework presented in this paper as a base for investigating role changes in other sectors.

## Conclusion

The role of the health care professional has shifted from a task-oriented approach to a relation-oriented approach, and brings along with it a new set of obstacles, needs, and competences that needs to be addressed to allow for a smooth transition.

## Author contributions

AvS outlined and executed the methodology of the article, and was responsible for writing the abstract, introduction and parts of the discussion section, rewriting and integrating the conclusions made by the other authors in the main body, as well as formulating new conclusions in the process. Contributed to revising and final approval. JvW was responsible for assisting in the execution of the methodology, writing various parts of the main body, parts of the discussion section, and providing the initial conceptualization of the review. Contributed to revising and final approval. IK was responsible for assisting in the execution of the methodology, for writing various parts of the main body, parts of the discussion section. Contributed to revising and final approval.

### Conflict of interest statement

The authors declare that the research was conducted in the absence of any commercial or financial relationships that could be construed as a potential conflict of interest.

## Appendix

### MMAT-criteria used in this review, adopted from pluye et al. (2011, p. 1).

**Table:**

Screening questions (for all types of studies)	Are there clear qualitative and quantitative research questions (or objectives), or a clear mixed methods question (or objective)? Do the collected data allow address the research question (objective)? E.g., consider whether the follow-up period is long enough for the outcome to occur (for longitudinal studies or study components).
1. Qualitative studies	1.1. Are the sources of qualitative data (archives, documents, informants, observations) relevant to address the research question (objective)? 1.2. Is the process for analyzing qualitative data relevant to address the research question (objective)? 1.3. Is appropriate consideration given to how findings relate to the context, e.g., the setting, in which the data were collected? 1.4. Is appropriate consideration given to how findings relate to researchers' influence, e.g., through their interactions with participants?
2. Quantitative randomized controlled (trials) studies	2.1. Is there a clear description of the randomization (or an appropriate sequence generation)? 2.2. Is there a clear description of the allocation concealment (or blinding when applicable)? 2.3. Are there complete outcome data (80% or above)? 2.4. Is there low withdrawal/drop-out (below 20%)?
3. Quantitative nonrandomized studies	3.1. Are participants (organizations) recruited in a way that minimizes selection bias? 3.2. Are measurements appropriate (clear origin, or validity known, or standard instrument; and absence of contamination between groups when appropriate) regarding the exposure/intervention and outcomes? 3.3. In the groups being compared (exposed vs. non-exposed; with intervention vs. without; cases vs. controls), are the participants comparable, or do researchers take into account (control for) the difference between these groups? 3.4. Are there complete outcome data (80% or above), and, when applicable, an acceptable response rate (60% or above), or an acceptable follow-up rate for cohort studies (depending on the duration of follow-up)?
4. Quantitative studies	4.1. Is the sampling strategy relevant to address the quantitative research question (quantitative aspect of the mixed methods question)? 4.2. Is the sample representative of the population understudy? 4.3. Are measurements appropriate (clear origin, or validity known, or standard instrument)? 4.4. Is there an acceptable response rate (60% or above)?
5. Mixed methods studies	5.1. Is the mixed methods research design relevant to address the qualitative and quantitative research questions (or objectives), or the qualitative and quantitative aspects of the mixed methods question (or objective)? 5.2. Is the integration of qualitative and quantitative data (or results^*^) relevant to address the research question (objective)? 5.3. Is appropriate consideration given to the limitations associated with this integration, e.g., the divergence of qualitative and quantitative data (or results^*^) in a triangulation design?
